# Schwannome étage du nerf médian: à propos d'un cas

**DOI:** 10.11604/pamj.2014.19.3.3239

**Published:** 2014-09-01

**Authors:** Jalal Boukhris, Mostapha Boussouga, Abdelouahab Jaafar, Belkacem Chagar

**Affiliations:** 1 Service de Traumatologie Orthopedie II, Hmimv Rabat, Maroc

**Keywords:** Schwannome, nerf médian, énucléation chirurgicale, Schwannoma, median nerve, surgical enucleation

## Abstract

Les schwannomes bénins sont les plus fréquentes des tumeurs nerveuses. En règle isolées, le caractère étagé de ces tumeurs reste exceptionnel. Ces tumeurs surviennent avec prédilection chez l'adulte de 20 à 50 ans, et toujours indifféremment l'homme et la femme. Le délai d'apparition des premiers signes est généralement long. L'imagerie par résonance magnétique permet d'orienter le diagnostic mais c'est l'histologie qui le confirme. Le traitement repose essentiellement sur l’énucléation chirurgicale. L’évolution est généralement favorable. Nous rapportons un cas rare de schwanome étagé développé au dépend du nerf médian, en détaillant les aspects diagnostic, thérapeutiques et évolutifs à travers une revue de la littérature récente.

## Introduction

Les tumeurs primitives des nerfs périphériques représentent 1 à 2% des tumeurs des tissus mous. Il convient de distinguer le schwannomes bénin et le neurofibrome des tumeurs malignes survenant généralement au cours d'une maladie de Recklinghausen. Nous rapportons un cas rare de schwannome étagé développé aux dépens du nerf médian. L'origine nerveuse de la tumeur ayant été suspectée en préopératoire sur ses caractéristiques cliniques et confronté aux données de l'imagerie par résonance magnétique.

## Patient et observation

Patiente de 23 ans, sans antécédents pathologiques particuliers, présentant depuis trois mois une tuméfaction de la face antérieure de l'avant bras gauche augmentant progressivement de volume avec des paresthésies et des décharges électriques dans le territoire du nerf médian. L'examen clinique local a noté une masse de 5 cm de diamètre, fixe par rapport au plan profond, l'examen loco-régional a objectivé la présence de troubles sensitif dans le territoire du nerf médian à type de fourmillements et paresthésies, l'examen général était sans particularité.

**Figure 1:**
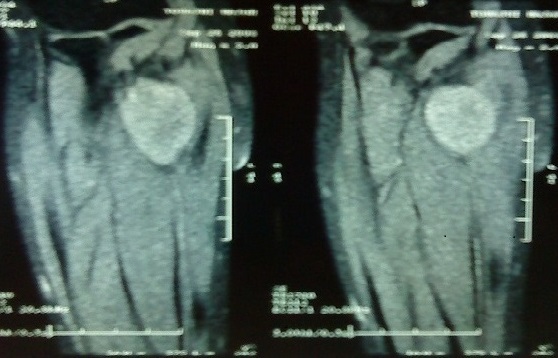
Coupe IRM en séquence pondérée T1 montrant une tumeur de même signal que le tissu musculaire avoisinant

**Figure 2:**
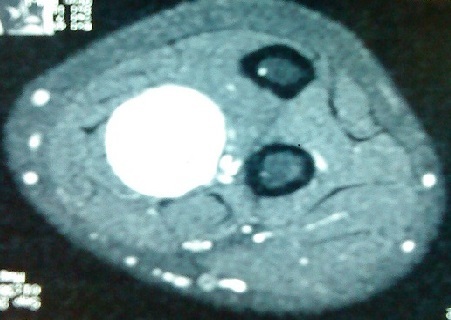
Coupe IRM en séquence pondérée T2 montrant la tumeur de signal très intense avec quelques plages centrale d'hyposignal

**Figure 3:**
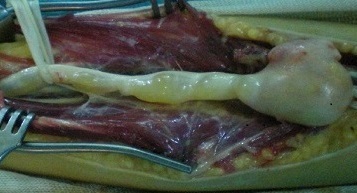
Individualisation de la tumeur et mise sur lac du nerf median

**Figure 4:**
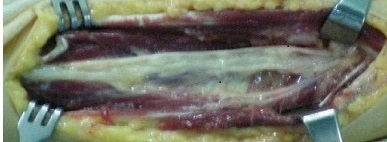
L'aspect per-opératoire après exérèse de la tumeur

La radiographie standard de l'avant bras était normale, le bilan paraclinique a été complété par un EMG qui n'a pas révélé d'anomalies et une IRM qui a mis en évidence tumeur de même signal que le tissu musculaire sur les séquences pondérées en T1 (Figure 1) et de signal très intense en T2 avec quelques plages centrales d'hyposignal (Figure 2). La patiente a bénéficié d'une énucléation chirurgicale de la tumeur (Figure 3), l’‘examen anatomo-pathologique de la pièce opératoire (Figure 4) a confirmé le diagnostic de schwanome bénin. A un an de recul, aucune récidive n'a été notée, les paresthésies ont disparu et la patiente est très satisfaite.

## Discussion

Les schwannomes bénins; autrefois appelés neurinomes, sont les plus fréquentes des tumeurs nerveuses [1]. Ils se développent aux dépens de cellules de Schwann, formant une prolifération macroscopiquement lisse, arrondie, jaunâtre et encapsulée [1, 2]. Ils sont facilement clivables des faisceaux nerveux qu'ils refoulent au lieu de les envahir; permettant ainsi une énucléation complète de la tumeur [1, 2]. La transformation maligne est exceptionnelle voire discutée, elle surviendrait principalement dans le cadre d'une maladie de Recklinghausen [2]. Ces tumeurs surviennent avec prédilection chez l'adulte de 20 à 50 ans, et toujours indifféremment l'homme et la femme. Elles se localisent préférentiellement à la face antérieure des membres supérieurs, classiquement au niveau des grands troncs nerveux [3, 4]. Il faut savoir évoquer le diagnostic de schwannome bénin devant une douleur ou des paresthésies d'un membre supérieur sans anomalie clinique évidente [3, 5, 6]. Les schwannomes sont en règle des tumeurs isolées de taille modérée et de croissance lente, palpables lorsqu'ils sont volumineux ou superficiels. Le caractère étagé de ces tumeurs reste exceptionnel [7]. Le délai d'apparition des premiers signes est généralement long; souvent plusieurs années [7]. Les douleurs à type de paresthésie sont souvent les premières et uniques manifestations [6] comme le cas de notre patiente. Les déficits sensitifs et moteurs objectifs sont rares en raison du caractère non infiltrant de la tumeur, ce qui explique que l'exploration électromyographique soit généralement normale [6]. L'IRM permet d'orienter le diagnostic en mettant en évidence une tumeur de même signal que le tissu musculaire sur les séquences pondérées en T1 et de signal très intense en T2 avec quelques plages centrales d'hyposignal, mais elle ne permet pas de différencier les schwannomes des neurofibromes [2–6], c'est l'histologie qui permet de confirmer le diagnostic [1, 2, 5, 6]. Histologiquement, le neurofibrome solitaire représente le principal diagnostic différentiel [1–6]. Le traitement idéal de ces tumeurs consiste en une énucléation chirurgicale avec dissection soigneuse des faisceaux nerveux avoisinants [3, 5, 6, 8] comme ce fût le cas de notre patiente; cependant la simple résection de la tumeur avec son nerf d'origine est parfois possible en cas de localisation distale sur un nerf sensitif superficiel [8]. L’évolution est généralement favorable après résection chirurgicale

## Conclusion

Les schwannomes sont en règle des tumeurs bénignes isolées. Le caractère étagé de ces tumeurs est exceptionnelle. L'imagerie par résonance magnétique permet d'orienter le diagnostic mais c'est l'histologie qui le confirme. Le traitement idéal de ces tumeurs consiste en une énucléation chirurgicale avec dissection soigneuse des faisceaux nerveux avoisinants. L’évolution est généralement favorable.
